# Agonist-induced phosphorylation of orthologues of the orphan receptor GPR35 functions as an activation sensor

**DOI:** 10.1016/j.jbc.2022.101655

**Published:** 2022-01-29

**Authors:** Nina Divorty, Laura Jenkins, Amlan Ganguly, Adrian J. Butcher, Brian D. Hudson, Stefan Schulz, Andrew B. Tobin, Stuart A. Nicklin, Graeme Milligan

**Affiliations:** 1 The Centre for Translational Pharmacology, Institute of Molecular, Cell and Systems Biology, College of Medical, Veterinary and Life Sciences, University of Glasgow, Glasgow, United Kingdom; 2 Institute of Cardiovascular and Medical Sciences, College of Medical, Veterinary and Life Sciences, University of Glasgow, Glasgow, United Kingdom; 3 Department of Clinical Neurosciences, University of Cambridge, Cambridge, United Kingdom; 4 7TM Antibodies GmbH, Jena, Germany; 5 Institute of Pharmacology and Toxicology, University Hospital Jena, Jena, Germany

**Keywords:** GPCR, arrestin, phosphorylation, phospho-site–specific antisera, GPR35, λ-PPase, lambda protein phosphatase, BRET, bioluminescence resonance energy transfer, BSA, bovine serum albumin, eYFP, enhanced yellow fluorescent protein, GPCR, G protein-coupled receptor, GPR35, G protein–coupled receptor 35, GRK, G protein-coupled receptor kinase, HBSS, Hanks' buffered saline solution, hGPR35, human GPR35, mGPR35, mouse GPR35, PDM, phosphorylation-deficient mutant, PEI, polyethylenimine

## Abstract

G protein-coupled receptor 35 (GPR35) is poorly characterized but nevertheless has been revealed to have diverse roles in areas including lower gut inflammation and pain. The development of novel reagents and tools will greatly enhance analysis of GPR35 functions in health and disease. Here, we used mass spectrometry, mutagenesis, and [^32^P] orthophosphate labeling to identify that all five hydroxy-amino acids in the C-terminal tail of human GPR35a became phosphorylated in response to agonist occupancy of the receptor and that, apart from Ser^294^, each of these contributed to interactions with arretin-3, which inhibits further G protein-coupled receptor signaling. We found that Ser^303^ was key to such interactions; the serine corresponding to human GPR35a residue 303 also played a dominant role in arrestin-3 interactions for both mouse and rat GPR35. We also demonstrated that fully phospho-site–deficient mutants of human GPR35a and mouse GPR35 failed to interact effectively with arrestin-3, and the human phospho-deficient variant was not internalized from the surface of cells in response to agonist treatment. Even in cells stably expressing species orthologues of GPR35, a substantial proportion of the expressed protein(s) was determined to be immature. Finally, phospho-site–specific antisera targeting the region encompassing Ser^303^ in human (Ser^301^ in mouse) GPR35a identified only the mature forms of GPR35 and provided effective sensors of the activation status of the receptors both in immunoblotting and immunocytochemical studies. Such antisera may be useful tools to evaluate target engagement in drug discovery and target validation programs.

Although classified as an orphan G protein-coupled receptor (GPCR) (1, 2), GPR35 has a rich pharmacology where, over time, a host of synthetic and naturally generated compounds have been shown to be capable of activating the receptor with potency ranging from modest to high (see (2, 3) for review). There has been growing interest in targeting GPR35 in a therapeutic context based, at least in part, on strong genetic association between single nucleotide polymorphic variants of human GPR35 and inflammatory diseases of the lower gut, including ulcerative colitis (2), as well as the high level of expression of the receptor in the colon and other regions of the intestine (2). Although there are differing views on the most appropriate modality of ligands that might effectively treat such diseases, currently the primary focus is on activating the receptor and hence on the identification and optimization of agonist ligands (2). Although a range of approaches have been used to identify agonists and antagonists of GPR35 (2), in the main, efforts to identify novel regulators from small molecule chemical libraries have been based on activator-induced interactions between the receptor and an arrestin isoform (4, 5, 6, 7). This reflects that, no matter the specific assay format employed, agonist-induced interactions between GPR35 orthologues and arrestin isoforms are generally extremely robust, whereas suitable G protein-based signaling assays have been more challenging to develop and implement (8).

A general concern in the development of therapeutic programs based on agonist ligands is the, at least theoretical, potential to induce desensitization or tachyphylaxis with sustained exposure to the ligand. Given the well-known roles of agonist-induced phosphorylation in arrestin interactions with many GPCRs (9, 10, 11, 12) and the roles of arrestins in receptor desensitization and internalization from the surface of cells (13) it is perhaps surprising that detailed analyses of the sites and mechanisms of agonist-regulated phosphorylation of GPR35, and how this might vary between human and rodent orthologues, has not been reported. Herein, we address this for each of human, mouse, and rat GPR35, using combinations of mass spectrometry, to define amino acids that become phosphorylated in an agonist-dependent manner and both [^32^P] incorporation and mutagenesis to define the contribution of these to arrestin-3 interactions. We then employ the information gained to design and develop phospho-site–specific antisera able to act as biosensors that can identify post-activation states of both human and mouse GPR35 in cells transfected to express the receptor orthologues.

The current studies provide a comprehensive analysis of how phosphorylation of GPR35 is achieved as well as novel reagents that will be of substantial value in further defining pathophysiological roles of GPR35 and the potential to target these productively.

## Results

**Figure 1:**
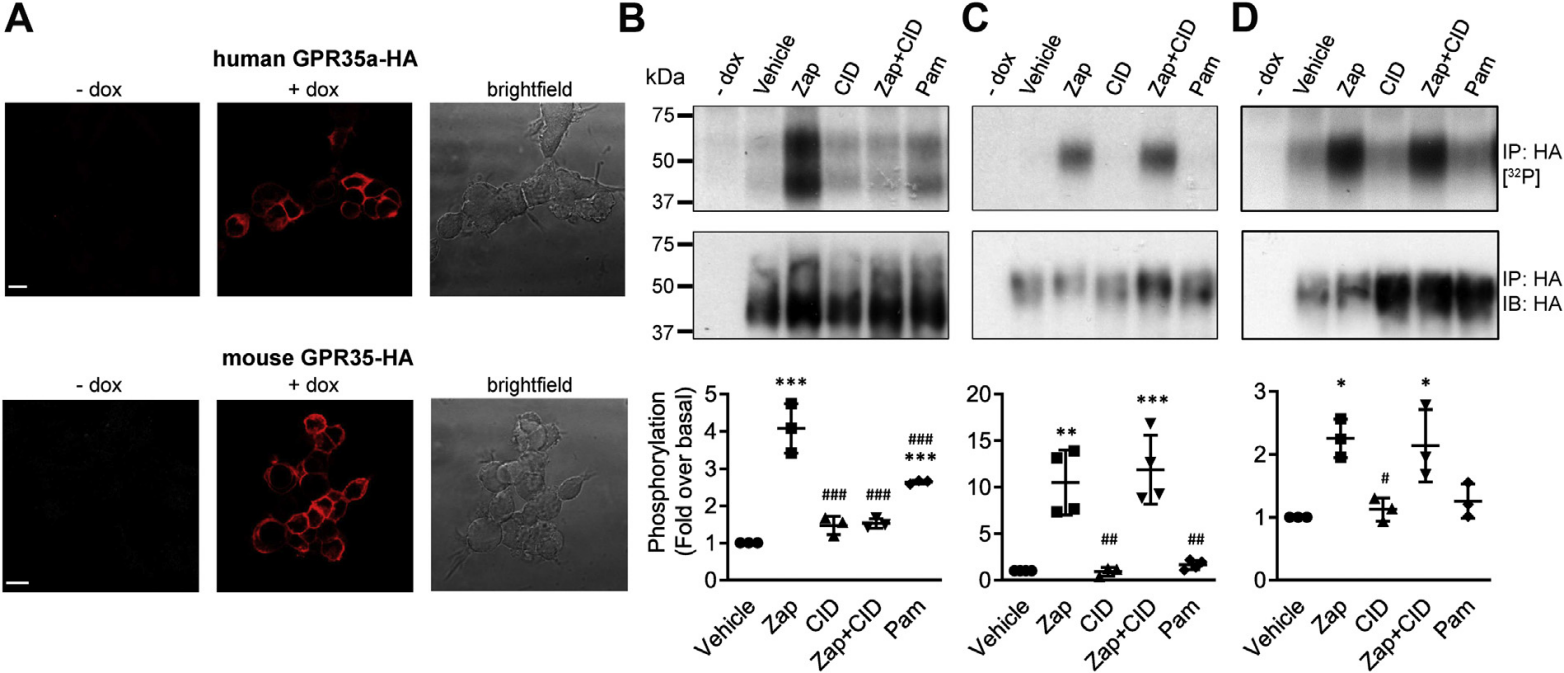
**Human GPR35a, mouse and rat GPR35 undergo agonist-dependent phosphorylation.***A*, images of anti-HA immunocytochemistry in cells harboring hGPR35a-HA (*upper panels*) or mGPR35-HA (*lower panels*) in the absence (−dox) and following doxycycline-induced (+dox) expression of the receptor constructs. Corresponding bright-field images (brightfield) are shown. Scale bar = 10 μm. Representative autoradiographs of [^32^P] (*top panels*) and anti-HA immunoblots [IB] (*center panels*) showing [^32^P] incorporation into (*B*) hGPR35a-HA, (*C*) mGPR35-HA, and (*D*) mGPR35-HA HA immunoprecipitates [IP]). Cells were treated with zaprinast (Zap) or pamoic acid (Pam) for 5 min prior to lysis. CID-2745687-treated cells (Zap+CID) were preincubated with CID-2745687 for 15 min prior to addition of Zap. Quantification (*lower panels*) shows mean fold change of [^32^P] incorporation over vehicle-treated cells, measured by densitometric analysis of autoradiographs from n = 3 independent experiments ± SD, ∗*p* < 0.05, ∗∗*p* < 0.01, ∗∗∗*p* < 0.001 compared with vehicle; #*p* < 0.05, ##*p* < 0.01, ###*p* <0.001 compared with zaprinast in one-way ANOVA with Tukey’s multiple comparisons posttest. (dox = doxycycline; Zap = 100 μM zaprinast; CID = 10 μM CID-2745687; Pam = 100 nM pamoic acid). GPR35, G protein-coupled receptor 35.

Across the GPCR superfamily receptor-arrestin interactions are frequently preceded by agonist-promoted phosphorylation of hydroxy-amino acids in the C-terminal tail of the receptor, the third intracellular loop, or both. To explore this for mouse GPR35 and its human equivalent splice variant GPR35a (2) we stably expressed C-terminally HA-epitope–tagged forms of either mouse GPR35 (mGPR35-HA) or human GPR35a (hGPR35a-HA) in Flp-In-TREx 293 cells. Following treatment of such cells with vehicle, or with doxycycline, which is anticipated to induce expression of the receptor constructs, we initially probed cells with anti-HA to confirm that expression was indeed induced for each orthologue (Fig. 1*A*). Subsequently we labeled vehicle-treated and doxycycline-induced cells with [^32^P] orthophosphate. Following subsequent exposure of the cells to compounds with known potency/affinity and/or species selectivity for mouse and human GPR35 (2, 14) the GPR35-HA orthologues were immunoprecipitated with anti-HA, resolved by SDS-PAGE, and incorporation of [^32^P] determined (Fig. 1, *B* and *C*). In cells in which either the receptor was not induced or those in which the construct was induced but which were not treated with an appropriate agonist, incorporation of [^32^P] was negligible for both hGPR35a-HA (Fig. 1*B*) and mGPR35-HA (Fig. 1*C*). Addition of zaprinast, which is known to be an activator with moderate but similar potency at human and mouse forms of GPR35 (15, 16, 17), promoted incorporation of [^32^P] into such HA-immunoprecipitates of each species orthologue. In the case of mGPR35-HA (Fig. 1*C*) and, in addition, also in a more limited set of studies using the rat orthologue of GPR35 (rGPR35-HA) (Fig. 1*D*), this was into a seemingly single species of apparent molecular mass (M_r_) just over 50 kDa, while for hGPR35a-HA, two distinct bands were observed of apparent M_r_ 55 and 43 kDa (Fig. 1*B*). Interestingly, parallel anti-HA immunoblots of such immunoprecipitates of hGPR35a-HA indicated that the more rapidly migrating 43 kDa species was markedly more abundant than the less rapidly migrating 55 kDa form, although incorporation of [^32^P] into the two forms was broadly equal (Fig. 1*B*). We will return to this apparent dichotomy later. In the case of hGPR35a-HA, incorporation of [^32^P] promoted by zaprinast into each form was lacking in the co-presence of the ligand CID-2745687 (4) (Fig. 1*B*). However, pretreatment with CID-2745687, a compound which although having high affinity at human GPR35 (18, 19) has no significant affinity at mouse or rat GPR35 (8, 18), did not prevent zaprinast-promoted incorporation of [^32^P] into either mGPR35-HA (Fig. 1*C*) or rGPR35-HA (Fig. 1*D*). Pamoic acid is a potent but partial agonist of hGPR35a (4, 16) but has much lower potency at mouse GPR35. Consistent with this, although pamoic acid also promoted phosphorylation of hGPR35a-HA, this was to a lesser extent than zaprinast (Fig. 1*B*), while at mGPR35-HA, 100 nM pamoic acid was without effect (Fig. 1*C*). Like mGPR35, the rat orthologue displays low potency for pamoic acid, and at 100 nM, this ligand also did not promote substantial phosphorylation of the rat receptor (Fig. 1*D*).

The observed multiple forms of hGPR35a-HA in such studies reflected differential N-glycosylation of the receptor protein because following treatment with N-glycosidase F to remove such carbohydrates, a single anti-HA immunoreactive band of some 32 kDa was observed (Fig. S1). mGPR35-HA was also N-glycosylated, as following treatment with N-glycosidase F, anti-HA immunoblotting also detected a single polypeptide of some 32 kDa corresponding to this construct (Fig. S1). In each case, this apparent M_r_ is consistent with the primary amino acid sequence of the orthologues (hGPR35a = 309 amino acids, mGPR35 = 307 amino acids).

**Figure 2:**
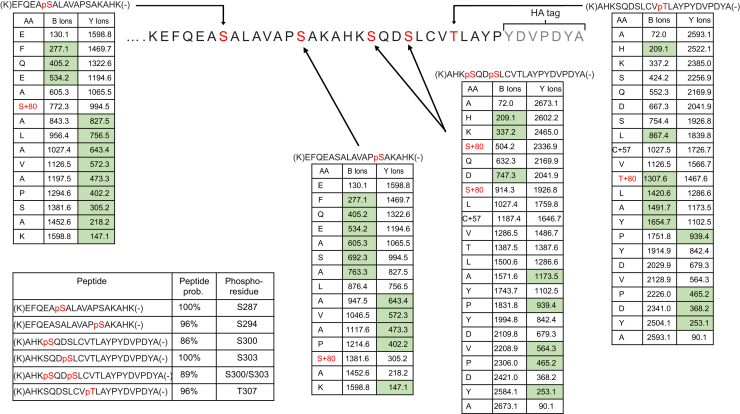
**Zaprinast can promote phosphorylation of five distinct amino acids in the C-terminal tail of human GPR35a.** LC-MS/MS identified five phosphorylation sites in the hGPR35a C-terminal tail following zaprinast stimulation. Composite outcomes of a series of independent experiments are combined. Fragmentation tables associated with phosphorylated peptides. All theoretical ions are shown, and those identified in the analysis are highlighted in *green*. Phosphorylated residues are highlighted in *red*. Peptide prob. indicates percentage probability of a correct peptide based on the discriminant score; both are generated by Scaffold software. The HA-epitope tag sequence within hGPR35a-HA is noted. See Figs. S2–S6 for further details. GPR35, G protein-coupled receptor 35.

**Figure 3:**
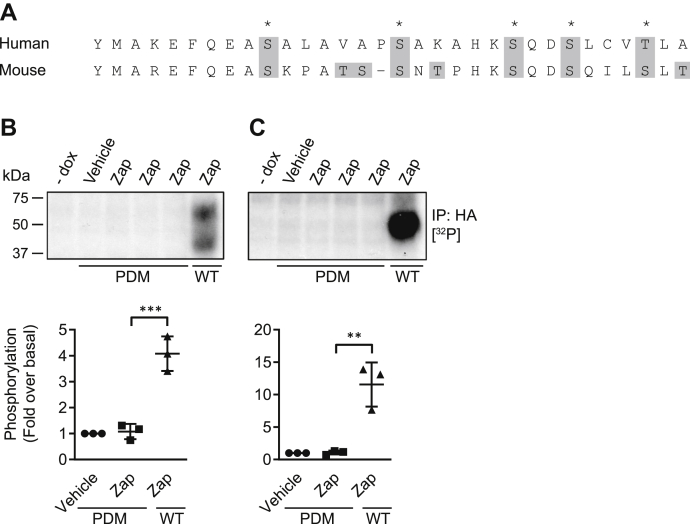
**Elimination of hydroxy-amino acids from the C-terminal tail of human and mouse GPR35 prevents zaprinast-induced phosphorylation.** C-terminal sequences of human (*upper*) and mouse (*lower*) GPR35 are presented in the one letter amino acid code. Amino acids highlighted and boxed in *gray* were mutated to alanine. *A*, residues marked with an *asterisk* are those found to be phosphorylated in LC-MS/MS performed on hGPR35a-HA. Representative autoradiographs showing [^32^P] incorporation into (*B*) human or (*C*) mouse WT or potentially phosphorylation-deficient mutants of GPR35 (PDM) are shown. Cells were stimulated with 100 μM zaprinast (Zap) for 5 min prior to lysis. Quantification (*lower panels*) shows fold change of [^32^P] incorporation over vehicle-treated cells, measured by densitometric analysis of autoradiographs from n = 3 independent experiments ± SD, ∗∗*p* < 0.01, ∗∗∗*p* < 0.001 in one-way ANOVA with Tukey’s multiple comparisons posttest. (dox = doxycycline.) GPR35, G protein-coupled receptor 35; PDM, phosphorylation-deficient mutant.

To attempt to identify the site or sites of agonist-induced phosphorylation, mass spectrometry was performed on tryptic peptides generated from affinity-purified hGPR35a-HA following treatment of cells with zaprinast (Fig. 2). Four distinct phospho-serines and a single phospho-threonine were identified in these studies, all located in the intracellular C-terminal tail region. These correspond to each of the hydroxy-amino acids present in this region (Figs. 2 and S2–S6 and Table S1). Notably, phosphorylation of Ser^300^ and Ser^303^, which are present in the same tryptic peptide, was observed both individually and in tandem in various experiments, indicating that multiple amino acids in hGPR35a-HA can be phosphorylated simultaneously. Serine and threonine residues present in the intracellular loops of hGPR35a were included in the peptide coverage from the mass spectrometry analysis, but no phosphorylation of these sites was observed. Thus, phosphorylation of hGPR35a appeared to occur exclusively within the C-terminal tail. To assess this directly, a potentially ‘phosphorylation-deficient’ mutant (PDM) of hGPR35a-HA was generated, in which each of the five identified sites of agonist-regulated phosphorylation was mutated to alanine (Fig. 3). We did not perform equivalent initial mass spectrometry studies on mGPR35. However, because the C-terminal tail of mGPR35 comprises a similar number of amino acids as hGPR35a, although in addition to the five hydroxy-amino acids that best align with those in the human sequence (Fig. 3*A*), there are four additional hydroxy-amino acids in the C-terminal region of mGPR35, we also generated a potential PDM of mGPR35 in which all nine hydroxy-amino acids in this region were converted to alanines (Fig. 3). These potential PDM constructs were also stably transfected to produce doxycycline-inducible Flp-In-TREx 293 cell lines. [^32^P] orthophosphate labeling followed by treatment with zaprinast and anti-HA immunoprecipitation from doxycycline-induced cells now failed to show incorporation of [^32^P] into either the human (Fig. 3*B*) or mouse (Fig. 3*C*) GPR35-PDM-HA constructs.

**Figure 4:**
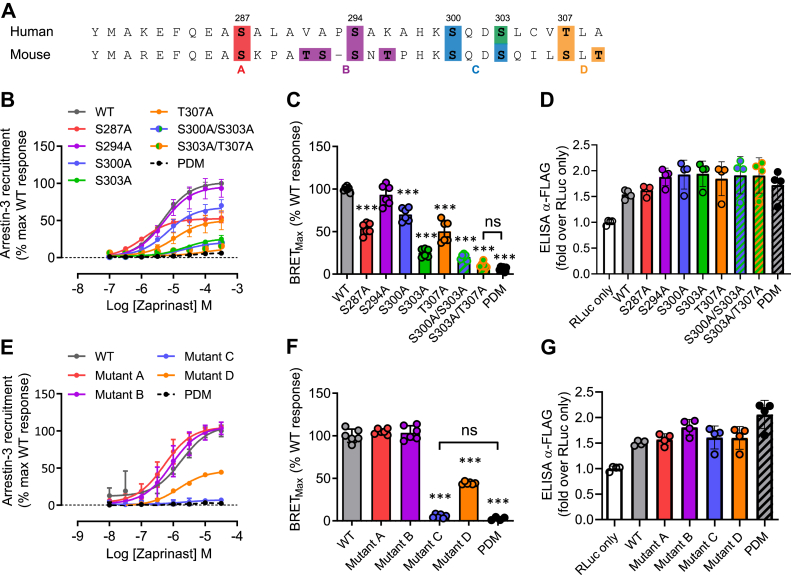
**Phosphorylation sites in the C-terminal tail of hGPR35a and mGPR35 are required for arrestin-3 recruitment induced by zaprinast.***A*, amino acid residues as highlighted (*colors*) were mutated to alanine in hGPR35a-eYFP and mGPR35-eYFP either individually or in the indicated combinations. *B*, zaprinast concentration–response curves for hGPR35a-YFP WT (*gray circles*); Ser^287^Ala (*red circles*); Ser^294^Ala (*purple circles*); Ser^300^Ala (*blue circles*); Ser^303^Ala (*green circles*); Thr^307^Ala (*orange circles*); Ser^300^Ala/Ser^303^Ala (*blue/green circles*); Ser^303^Ala/Thr^307^Ala (*green/orange circles*); and the phosphorylation-deficient mutant (*black circles*) in arrestin-3 interaction assay. *C*, maximal BRET stimulated by zaprinast treatment. *D*, anti-FLAG ELISA showing relative cell surface expression of FLAG-hGPR35-eYFP mutants co-transfected with arrestin-3–*Renilla* luciferase compared with cells transfected with arrestin-3–*Renilla* luciferase alone. *E*–*G*, studies equivalent to those in *B*–*D* were performed using mGPR35-eYFP. All data are pooled from n = 3 independent experiments performed in triplicate ± SD ∗∗∗*p* < 0.001 compared with WT; ns = not significant in one-way ANOVA with Tukey’s multiple comparisons posttest (shown for BRET_Max_ only). BRET, bioluminescence resonance energy transfer; eYFP, enhanced yellow fluorescent protein; GPR35, G protein-coupled receptor 35; mGPR35, mouse GPR35; PDM, phosphorylation-deficient mutant.

**Figure 5:**
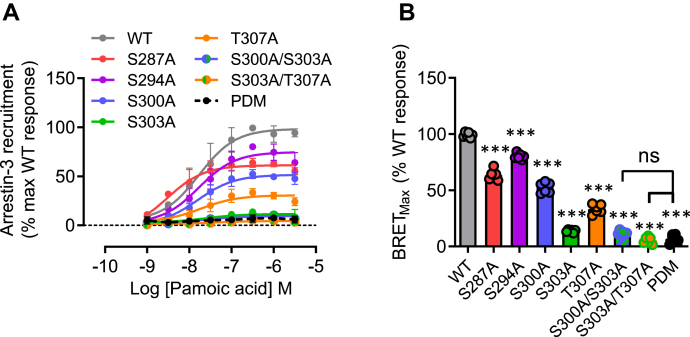
**Phosphorylation sites in the C-terminal tail of hGPR35a are required for arrestin-3 recruitment induced by pamoic acid.***A*, studies akin to those of Figure 4 were performed using pamoic acid as agonist: hGPR35a-YFP WT (*gray circles*); Ser^287^Ala (*red circles*); Ser^294^Ala (*purple circles*); Ser^300^Ala (*blue circles*); Ser^303^Ala (*green circles*); Thr^307^Ala (*orange circles*); Ser^300^Ala/Ser^303^Ala (*blue/green circles*); Ser^303^Ala/Thr^307^Ala (*green/orange circles*); and the phosphorylation-deficient mutant (*black circles*) were co-transfected with arrestin-3–*Renilla* luciferase and BRET studies performed after exposure to the indicted concentrations of pamoic acid. *B*, maximal BRET stimulated by pamoic acid treatment. All data are pooled from n = 3 independent experiments performed in triplicate ± SD ∗∗∗*p* < 0.001 compared with WT; ns = not significant in one-way ANOVA with Tukey’s multiple comparisons posttest. BRET, bioluminescence resonance energy transfer; PDM, phosphorylation-deficient mutant.

**Figure 6:**
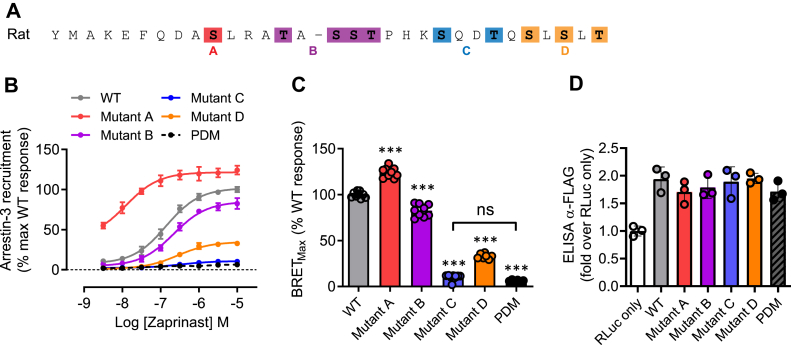
**Phosphorylation sites in the C-terminal tail of rat GPR35 are required for arrestin-3 recruitment induced by zaprinast.***A*, the C-terminal sequence of rat GPR35 is shown. Amino acid residues as highlighted (*colors*) were mutated to alanine in rGPR35-eYFP in the indicated combinations (*A*). *B*, zaprinast concentration–response curves for rGPR35-eYFP (*black circles*); a fully phospho-deficient form (PDM) (*black squares*) and the combinations of alanine mutations corresponding to *A*–*D* in panel (*A*), (*red*, *purple*, *blue*, *orange*, as noted in arrestin-3 interaction assays. *C*, maximal BRET stimulated by zaprinast treatment of the forms in (*B*). *D*, anti-FLAG ELISA showing relative cell surface expression of rGPR35-eYFP and the same mutants co-transfected with arrestin-3–*Renilla* luciferase compared with cells transfected with arrestin-3–*Renilla* luciferase alone. All data are pooled from n = 3 independent experiments performed in triplicate ± SD, ∗∗∗*p* < 0.001 compared with WT; ns = not significant in one-way ANOVA with Tukey’s multiple comparisons posttest (shown for BRET_Max_ only). ∗∗*p* < 0.01, ∗∗∗*p* < 0.001 in one-way ANOVA with Tukey’s multiple comparisons posttest. (dox = doxycycline). BRET, bioluminescence resonance energy transfer; eYFP, enhanced yellow fluorescent protein; GPR35, G protein-coupled receptor 35; PDM, phosphorylation-deficient mutant.

To assess the contribution of sites of agonist-regulated phosphorylation to potential agonist-induced interactions with arrestins, we compared initially the ability of WT hGPR35a and hGPR35a–PDM to interact with arrestin-3 in a zaprinast-dependent manner. Following transient co-transfection into HEK293T cells of these two forms of hGPR35a, each with C-terminally attached enhanced yellow fluorescent protein (eYFP), and arrestin-3 linked to *Renilla* luciferase, zaprinast promoted bioluminescence resonance energy transfer (BRET) between hGPR35a-eYFP and arrestin-3–*Renilla* luciferase in a concentration-dependent manner (Fig. 4), but this was not observed for hGPR35a-PDM-eYFP (Fig. 4). Except for Ser^294^Ala, individual alanine replacement of each of the five hydroxy-amino acids (Ser^287^, Ser^294^, Ser^300^, Ser^303^, Thr^307^) in the C-terminal tail of hGPR35a-eYFP (Fig. 4*A*), resulted in reduced maximal zaprinast-stimulated BRET signals (Fig. 4, *B* and *C*) with no substantial effects on agonist potency. Double replacement of either Ser^300^ and Ser^303^ or Ser^303^ and Thr^307^ resulted in almost complete ablation of interactions (Fig. 4, *B* and *C*). This was despite the cell surface expression levels of each mutant being very similar to WT hGPR35a-eYFP as defined by intact cell ELISA assays that detected a FLAG-epitope tag engineered into the extracellular N-terminal domain of each construct (Fig. 4*D*). A highly similar pattern was observed when using the partial agonist pamoic acid (Fig. 5). mGPR35-PDM-eYFP, containing replacement by alanine of all nine hydroxy-amino acids in the C-terminal tail (Fig. 4*A*), also failed to recruit arrestin-3–*Renilla* luciferase in response to zaprinast (Fig. 4, *E* and *F*). Owing to the larger number of hydroxy-amino acids in the C-terminal tail of mGPR35, rather than assessing individual point mutants, we grouped these into four sets for mutational studies (Fig. 4*A*). The mutant containing alterations of residues equivalent to Ser^300^ and Ser^303^ in hGPR35a (Ser^298^ and Ser^301^ in mouse, [mutant C]) also failed to interact with arrestin-3–*Renilla* luciferase (Fig. 4, *E* and *F*). However, the other mutants were less impaired or even unaffected in this response (Fig. 4, *E* and *F*). Once again each of the mGPR35 mutants was delivered to the cell surface as effectively as the WT sequence (Fig. 4*G*). The rat sequence is similar to mouse in this region but with one further additional hydroxy-amino acid. We therefore adopted the strategy of producing four mutants covering the same regions as in mGPR35. The outcomes were similar to those observed for mGPR35 with the fully phospho-deficient mutant unable to interact with arrestin-3 in an agonist-dependent manner, while the mutant based on the pair of hydroxy-amino acids corresponding to Ser^300^ and Ser^303^ in hGPR35a (Ser^297^ and Thr^300^ in rat, [mutant C]) produced as large an effect on arrestin-3 recruitment as the fully phospho-deficient form (Fig. 6). In this case, combined mutation to alanine of the three most C-terminal hydroxy-amino acids also generated a form of the receptor with very limited interactions with arrestin-3 (Fig. 6).

**Figure 7:**
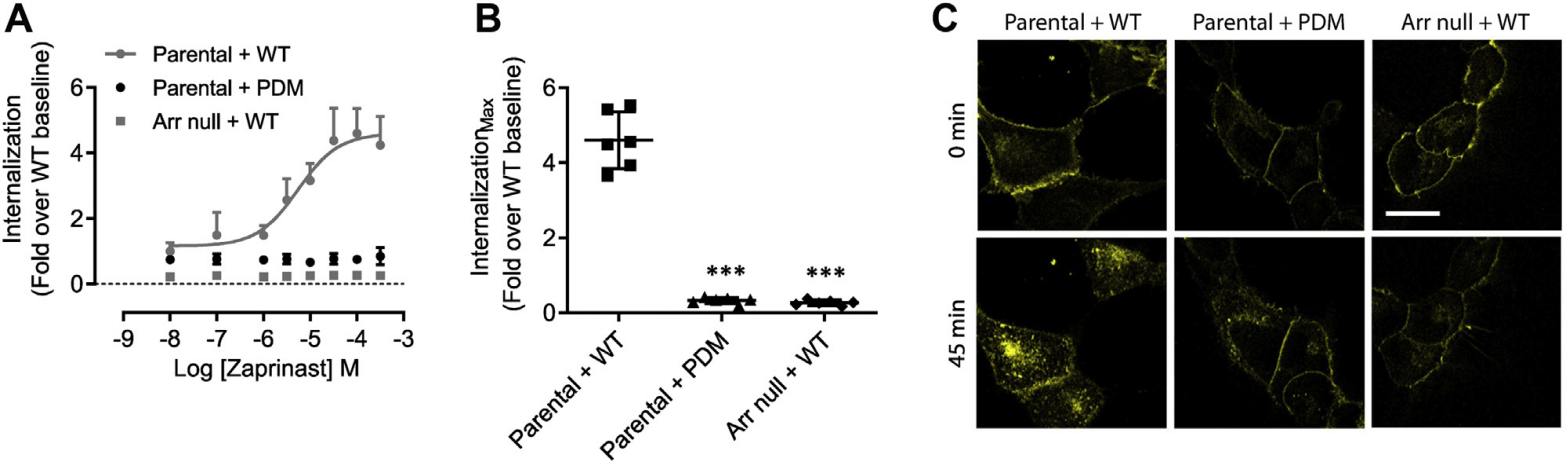
**Agonist-induced internalization of hGPR35a requires phosphorylation and an arrestin.***A*, hGPR35a-eYFP and hGPR35a-PDM-eYFP internalization was measured after treatment for 45 min with varying concentration of zaprinast in parental HEK293 cells (parental) stably expressing each construct or of hGPR35a-eYFP in HEK293 cells genome-edited to lack expression of both arrestin-2 and arrestin-3 (Arr null) (Ref (13)). *B*, maximal effect in *A*. Data are pooled from n = 3 independent experiments performed in triplicate ± SD; ∗∗∗*p* < 0.001 in one-way ANOVA. *C*, representative images of such studies. WT = GPR35 WT; PDM = GPR35 phosphorylation-deficient mutant; Arr = arrestin. Scale bar = 20 μm. eYFP, enhanced yellow fluorescent protein; GPR35, G protein-coupled receptor 35.

Receptor phosphorylation is frequently associated with subsequent arrestin-dependent internalization of the receptor from the cell surface. As shown previously (18) zaprinast, in a concentration-dependent manner, promoted internalization of hGPR35a-eYFP in HEK293 cells stably expressing this construct (Fig. 7). This effect of zaprinast was not reproduced in equivalent cells stably expressing hGPR35a-PDM-eYFP (Fig. 7), and as anticipated from the foregoing, zaprinast was unable to promote internalization of hGPR35a-eYFP expressed stably in genome-edited HEK293 cells that lack expression of both arrestin-2 and arrestin-3 (13, 20) (Fig. 7).

**Figure 8:**
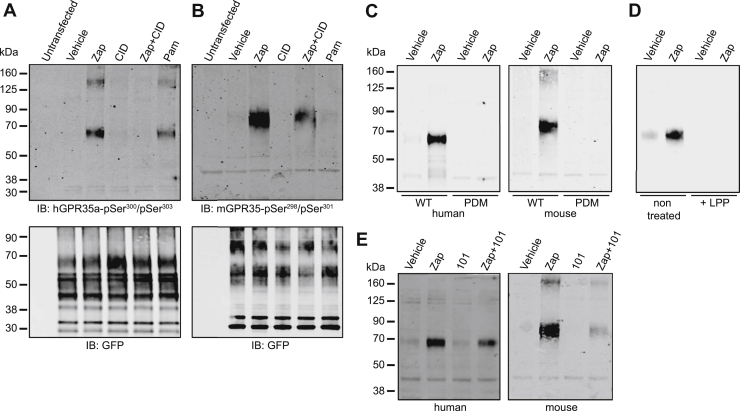
**Production and characterization of GPR35 phospho-site–specific antisera.** Peptides as described in the text and experimental procedures were used to generate immune responses in rabbits. These antisera were then used in immunoblots of lysates of HEK293T cells either nontransfected or transfected to express (*A*) hGPR35a-eYFP or (*B*) mGPR35-eYFP. Prior to production of lysates, cells were treated with the same range of ligands and combinations as in Figure 1. Antisera used were hGPR35a-pSer^300^/pSer^303^ (*A*) or mGPR35-pSer^298^/pSer^301^ (*B*). Further studies were performed on lysates of cells expressing hGPR35a-eYFP (*C*–*E*), hGPR35a-PDM-eYFP (*C*), mGPR35-eYFP (*C* and *E*), or mGPR35-PDM-eYFP (*C*) in which cells were pretreated with vehicle, zaprinast (Zap), compound 101 (101), or a combination of zaprinast and compound 101. In (*D*), the indicated samples were treated with Lambda protein phosphatase prior to separation by SDS-PAGE. Representative immunoblots are shown. eYFP, enhanced yellow fluorescent protein; GPR35, G protein-coupled receptor 35; LPP, Lambda protein phosphatase; mGPR35, mouse GPR35; PDM, phosphorylation-deficient mutant.

**Figure 9:**
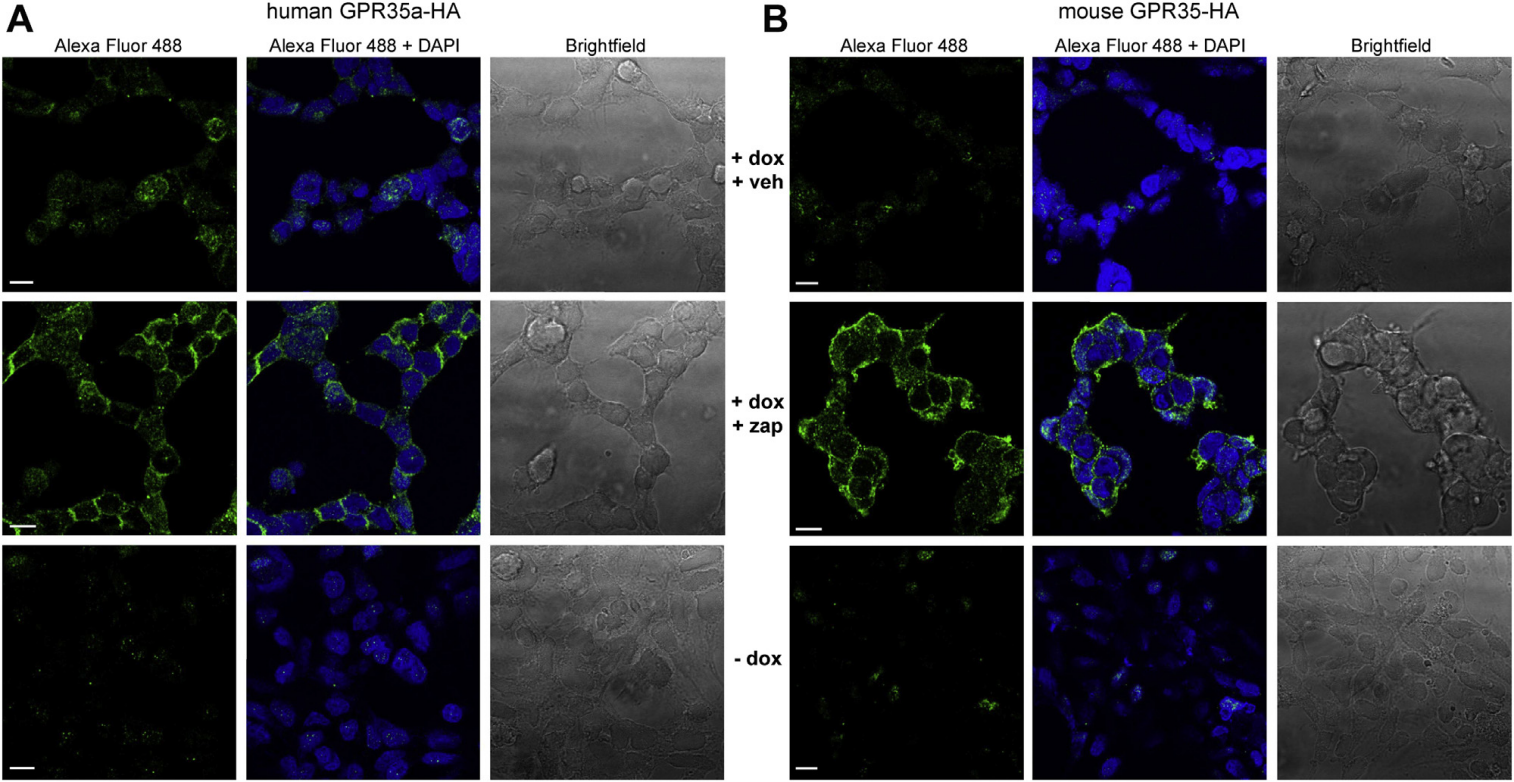
**GPR35 phospho-site–specific antisera function as biosensors of agonist activated, fully mature GPR35.** Cells as in Figure 1*A* able to express hGPR35a-HA (*A*) or mGPR35-HA (*B*) were either uninduced (−dox) or induced by treatment with doxycycline (+dox) and then treated with either vehicle (veh) or zaprinast (zap). Such cells were then used in immunocytochemical studies employing hGPR35a-pSer^300^/pSer^303^ or mGPR35-pSer^298^/pSer^301^ (*left panels*) (Alexa Fluor 488). Samples were counterstained with the nuclear dye DAPI (*center panels*). Brightfield images (*right panels*) are also shown. Scale bar = 10 μm. Representative images are shown. mGPR35, mouse GPR35.

**Figure 10:**
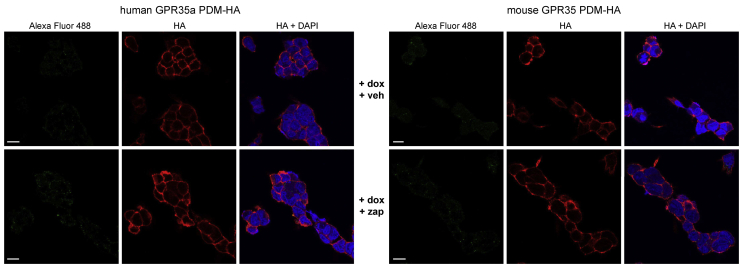
**Phospho-site–specific antisera fail to identify hGPR35a-PDM-HA or mGPR35-PDM-HA in immunocytochemical studies.** In experiments akin to those of Figure 9, Flp-In-TREx 293 cells harboring hGPR35a-PDM-HA (*left panels*) or mGPR35-PDM-HA (*right panels*) were induced to express the receptor by pretreatment with doxycycline (+dox) and then with either zaparinast (zap) or vehicle (veh). Immunocytochemical studies employed hGPR35a-pSer^300^/pSer^303^ (Alexa Fluor 488) and anti-HA (HA) (*left panels*) or mGPR35-pSer^298^/pSer^301^ (Alexa Fluor 488) and anti-HA (HA) (*right panels*). Samples were counterstained with the nuclear dye DAPI (DAPI). Scale bar = 10 μm. Representative images are shown. mGPR35, mouse GPR35; PDM, phosphorylation-deficient mutant.

With the aspiration of detecting the activation status of GPR35 *in situ*, we next turned to the production of antisera potentially able to selectively identify phosphorylated forms of GPR35. Based on the results above, we used a peptide from hGPR35a that incorporated both pSer^300^/pSer^303^ (KAHKpSQDpSLCVTL) to immunize rabbits. Following affinity purification, we then used such antisera to probe samples derived from vehicle and zaprinast-stimulated HEK293T cells that, as in the arrestin-3 interaction studies, had been transiently transfected to express hGPR35a-eYFP or not. Following capture of the receptor construct *via* a GFP-trap, immunoblotting with an anti-GFP antiserum showed the presence of a number of polypeptides that were absent in samples isolated from untransfected cells (Fig. 8*A*). Significant among these was a smear of GFP-immunoreactivity in the region of 70 kDa, as well as a range of more rapidly migrating forms (Fig. 8*A*). Parallel immunoblotting of such samples with the hGPR35a-pSer^300^/pSer^303^ antibodies identified the approx. 70 kDa polypeptide(s) and did so in a manner that was dependent on pretreatment of the cells with zaprinast (Fig. 8*A*), as the antiserum did not identify this polypeptide(s) in vehicle-treated cells (Fig. 8*A*). However, the hGPR35a-pSer^300^/pSer^303^ antibodies did not identify the more rapidly migrating polypeptides identified by the anti-GFP antiserum (Fig. 8*A*). As anticipated from the direct phosphorylation studies (Fig. 1), treatment of such transiently transfected cells with the human-specific GPR35 antagonist CID-2745687 did not promote phosphorylation of pSer^300^/pSer^303^ within hGPR35a-eYFP as assessed by lack of immunodetection with the hGPR35a-pSer^300^/pSer^303^ antibodies (Fig. 8*A*). However, pre-addition of CID-2745687 prevented this effect of zaprinast (Fig. 8*A*). Moreover, pamoic acid, although able to promote recognition of hGPR35a-eYFP by hGPR35a-pSer^300^/pSer^303^ antibodies, was again a partial agonist compared to zaprinast at this endpoint (Fig. 8*A*). In parallel with production of the hGPR35a-pSer^300^/pSer^303^ antibodies, we employed the equivalent peptide sequence from mGPR35 (TPHKpSQDpSQILSLT) to generate mGPR35-pSer^298^/pSer^301^-directed antibodies. Following transient expression of mGPR35-eYFP in HEK293T cells, we performed experiments akin to those above. Here, immunoblotting with anti-GFP again detected distinct sets of GFP-positive polypeptides, with a set centered at M_r_ approx. 75 kDa and a group of more rapidly migrating polypeptides (Fig. 8*B*). Once more, identification of these forms of mGPR35 with the mGPR35-pSer^298^/pSer^301^ antiserum was restricted to the higher M_r_ polypeptides (Fig. 8*B*), and such detection required that the cells had been exposed to zaprinast (Fig. 8*B*). As anticipated from the known pharmacology and species selectivity of CID-2745687, pre-addition of this compound did not block the effect of zaprinast at mGPR35-eYFP (Fig. 8*B*). That these interactions truly reflected agonist-induced phosphorylation of both the human and mouse orthologues of the receptor was evident in that no such immunoreactivity was observed when equivalent experiments were performed in cells induced to express hGPR35a-PDM-eYFP or mGPR35-PDM-eYFP (Fig. 8*C*). Moreover, treatment of agonist-stimulated hGPR35a-eYFP after capture on GFP-trap with Lambda protein phosphatase (λ-PPase) to remove phosphate from the protein eliminated identification of the receptor construct by the hGPR35a-pSer^300^/pSer^303^ antibodies confirming that they were indeed identifying phosphorylation of pSer^300^, pSer^303^, or both sites (Fig. 8*D*). In many settings, agonist-induced phosphorylation of GPCRs reflects the action of one or more members of the G protein-coupled receptor kinase (GRK) family (21, 22). Pretreatment of cells with the GRK2/3 inhibitor compound 101 (23, 24) substantially reduced zaprinast-induced binding by these antisera of both the human and mouse orthologues (Fig. 8*E*), implying an important role for GRK2 and/or GRK3. As an extension to these studies, in the Flp-In TREx 293 cells harboring either hGPR35a-HA or mGPR35-HA at the Flp-In TREx locus (25), we performed immunocytochemical studies using either the hGPR35a-pSer^300^/pSer^303^ or mGPR35-pSer^298^/pSer^301^ antisera. Specific signal, located at both the cell surface and in punctate intracellular vesicles, was observed largely when either receptor construct had been expressed, and the cells had been exposed to zaprinast (Fig. 9). In cells induced to express hGPR35a-HA, hGPR35a-pSer^300^/pSer^303^ antibodies indicated a level of detection in the absence of zaprinast that may imply a degree of constitutive phosphorylation of the receptor in this setting (Fig. 9). As anticipated from the lack of identification of either hGPR35a-PDM or mGPR35-PDM in the immunoblotting studies, these antisera also failed to recognize hGPR35a-PDM-HA or mGPR35-PDM-HA in immunocytochemical studies in Flp-In TREx 293 cells induced to express either of these constructs (Fig. 10).

## Discussion

Although it is well established that agonist-induced phosphorylation is a key step in promoting interactions between an agonist-occupied GPCR and isoforms of arrestin (26, 27, 28), it remains uncommon to have a comprehensive map of the identity of specific sites of such posttranslational regulation and the extent to which each modified amino acid may contribute to the effect. Herein, we report on both these topics for GPR35 and use this information to generate phospho-site–specific antisera that act as activation state–specific biosensors. While nominally an ‘orphan’ receptor, many ligands, both endogenously produced and synthetic, are known to be able to activate GPR35, and this receptor is attracting considerable interest as a potential therapeutic target (2, 3). The strong link between sequence variants of GPR35 and various inflammatory diseases of the lower gut has focused attention on inflammatory bowel diseases, but a wide range of other areas are also being considered (2).

Although there are certainly distinct, non–G protein-dependent signaling cascades engaged by GPCRs subsequent to interacting with an arrestin (27), such interactions have traditionally been viewed as the means to terminate G protein-dependent signaling. In efforts to develop agonist-based GPCR therapeutic programs, understanding of the potential for desensitization may be important to optimize target coverage over time in lead optimization work. Moreover, biased ligands, able to selectively promote G protein activation or arrestin engagement, have been described for many GPCRs (28, 29, 30, 31). As such, detailed understanding of the molecular basis and consequences of arrestin interactions with individual GPCRs is an intrinsically important goal, while detection of such events in cells and tissues can potentially provide a biosensor of current and prior GPCR occupancy by an activating ligand.

To define the sites of agonist (zaprinast)-induced phosphorylation in hGPR35a, we employed mass spectrometry on tryptic digests of the immunoprecipitated HA-tagged receptor. Here, we observed that each hydroxy-amino acid in the intracellular C-terminal tail of the receptor was subject to modification. Thus, in common with many other GPCRs (32, 33), GPR35 is multiply phosphorylated in clusters of Ser/Thr in the C-terminal tail. By making initial single point mutants, conversion of each of Ser^287^, Ser^300^, Ser^303^, and Thr^307^ to alanine markedly reduced the effectiveness of zaprinast-induced arrestin-3 interactions. In this way, GPR35 fits a general pattern of the importance of receptor phosphorylation for the recruitment of arrestins where those receptors that are primarily phosphorylated on residues in the C-tail, *e.g.*, free fatty acid receptor 4 (34, 35, 36), the vasopressin V2 receptor (37, 38), ghrelin (39) and the parathyroid hormone receptor (40) show higher dependency on phosphorylation for the recruitment of arrestins than receptors whose phosphorylation sites lie within the third intracellular loop (*e.g.*, M_1_ and M_3_-muscarinic receptors) (41, 42, 43, 44, 45). Interestingly, although all of the C-tail phosphorylation sites on hGPR35a, apart from Ser^294^, appear to play a combinatorial role in arrestin recruitment, one site in particular, Ser^303^, appeared to play a larger role than the others. This observation is consistent with the notion that different patterns of receptor phosphorylation may result in different signaling outcomes. This notion has been extended in what has been coined the ‘barcode’ hypothesis (32, 33) where different patterns of receptor phosphorylation exist in different cell/tissue types and that these can contribute to or initiate distinct signaling patterns (46, 47, 48). Although there is no direct structural information available on GPR35, the C-terminal tail is short. Moreover, although the BRET-based proximity assays we have employed do not report on affinity of interaction between the receptor and the arrestin, in general GPR35 gives a very large and robust signal in such assays compared to many other GPCRs we have tested. Others have tried to provide general rules for the basis of interactions between sites of phosphorylation in the C-terminal tail of GPCRs and arrestins, with particular focus on rhodopsin and a chimeric receptor with a β_2_-adrenoceptor core and the C-terminal tail of the vasopressin V_2_ receptor (49, 50). We observed a number of features in hGPR35a with similarity to these models. Firstly, we noted that no single hydroxy-amino acid completely prevented interactions with arrestin-3, although, as noted earlier, the Ser^303^Ala alteration produced the greatest individual effect. As with the models noted above (49, 50) key amino acids are separated by two or three others, and in addition, GPR35 has both a positively charged (Lys) and negatively charged (Asp) residue in close proximity to the key hydroxy-amino acids (49, 50). Although there are additional hydroxy-amino acids in both rat and mouse GPR35, residues equivalent to Ser^300^ and Ser^303^ were similarly important. This suggests the mode of interaction for GPR35 may well be similar to the ideas presented by Zhou *et al.* (50). However, it should be noted that we employed arrestin-3 herein, while in other studies, the partner was arrestin-2. It is also noteworthy that conversion of Ser^294^ to Ala in hGPR35a did not produce an effect on arrestin-3 interactions. This is of interest because Ser^294^ is the site of a common single nucleotide polymorphism where substitution for Arg is present in 48% of the population, and this variation has been associated with cardiovascular disease (51).

By demonstrating here the importance of Ser^303^ (and also Ser^300^) in arrestin recruitment to hGPR35a, as well as the equivalent amino acids in both mouse and rat GPR35, and with the generation of phospho-specific antibodies to this amino acid pair in the human receptor sequence and their equivalents in the mouse orthologue, we are now in a position to probe the possibility that in physiologically relevant settings, GPR35 might be differentially phosphorylated on these residues in a manner that may regulate the extent to which the receptor interacts with arrestin isoforms.

Throughout efforts to identify ligands that regulate GPR35, assays that monitor interactions between the receptor and an arrestin have been widely used (2). In large part, this reflects that G protein activation assays for GPR35 have been limited (8). This reflects the uncommon feature of GPR35 to interact with Gα_13_ with marked selectivity over other G protein subtypes (8), or instead, they have relied on the availability of ‘label-free’ dynamic mass redistribution measurements (52, 53) that are both relatively expensive to perform and require specific reader technology (54, 55). By contrast, a range of arrestin interaction assays are widely available and are extremely effective in measuring agonist occupancy of GPR35. Based on how extensively these methods have been used, we wished to understand the basis of agonist-induced GPR35–arrestin interactions at a molecular level. As part of this, we were also cognizant that distinct pharmacology has frequently been reported between human and rodent orthologues of GPR35 (2, 14). We therefore performed sets of studies with both human and mouse GPR35 in parallel and, in subsets of studies, also extended these to the rat orthologue.

The current studies provide a comprehensive analysis of how phosphorylation of GPR35 is achieved and novel reagents that will be of substantial value in further defining pathophysiological roles of GPR35.

## Experimental procedures

### Materials

Zaprinast, pamoic acid, CID-2745687, and compound 101 were obtained from Tocris Bioscience. All cell culture reagents were from Thermo Fisher Scientific. Polyethylenimine (PEI) [linear poly(vinyl alcohol) (MW-25000)] was from Polysciences. λ-PPase was from New England BioLabs. cOmplete EDTA-free Protease Inhibitor Cocktail, PhosSTOP Phosphatase Inhibitor Cocktail, and *N*-glycosidase F were from Roche Diagnostics

### Antibodies

The rabbit phospho-site–specific GPR35 antiserum pSer^300^/pSer^303^-hGPR35a (Cat number (7TM0102C), raised against the sequence KAHKpSQDpSLCVTL, and the pSer^298^/pSer^301^-mGPR35 antiserum (7TM0102B), raised against the sequence TPHKpSQDpSQILSLT, were developed in collaboration with 7TM Antibodies GmbH. IRDye 800CW donkey anti-rabbit IgG, IRDye 800CW donkey anti-goat IgG, and IRDye 800CW goat anti-rat IgG were from LI-COR Biosciences. Alexa Fluor 488-goat anti-rabbit IgG and Alexa Fluor 594-donkey anti-rat IgG were from Abcam. Mouse monoclonal anti-FLAG M2 was from Merck. Horseradish peroxidase anti-mouse (sheep) was from GE Healthcare. High affinity anti-HA (rat) and anti-HA affinity matrix were from Roche Diagnostics.

### Generation of constructs

Generation of FLAG-hGPR35a-eYFP, hGPR35a-HA, FLAG-mGPR35-eYFP, and mGPR35-HA have been described previously (8, 18).The Stratagene QuikChange method (Stratagene, Agilent Technologies) was used to introduce alterations into each of the above constructs to produce both point mutants and phospho-deficient variants. Primers utilized for mutagenesis were provided by MWG Operon. Sequencing was carried out to confirm the introduction of the alterations.

### Maintenance of cell lines

Human embryonic kidney (HEK) 293T cells were maintained in Dulbecco’s modified Eagle’s medium supplemented with 0.292 g/l L-glutamine, 1% penicillin/streptomycin mixture, and 10% heat-inactivated fetal bovine serum at 37 ^o^C in a 5% CO_2_ humidified atmosphere. Flp-In TREx 293 cells (Thermo Fisher) were maintained in Dulbecco's modified Eagle's medium without sodium pyruvate, supplemented with 10% fetal bovine serum, 1% penicillin/streptomycin mixture, and 10 μg/ml blasticidin at 37 °C in a 5% CO2 humidified atmosphere.

### Transient transfection of cell lines

PEI-mediated transient transfection was used as the default method of transient transfection. For a 10 cm^2^ culture dish, 5 μg of DNA was diluted in 250 μl 150 mM NaCl and mixed 1:1 with 250 μl 150 mM NaCl containing 30 μg PEI. The mixture was vortexed for 10 s and incubated for 10 min at room temperature before adding dropwise to the dish. This procedure was scaled down for 6-well plates. Cells were incubated with the PEI overnight at 37 °C, then transfection medium was replaced with fresh culture medium. Cells were incubated for a further 24 to 48 h before using in assays. For some studies where PEI transfection negatively affected cell morphology and assay reliability, cells were instead transiently transfected using Lipofectamine (Thermo Fisher) as per the manufacturer’s instructions. For a 6-well plate, 0.5 to 2.5 μg DNA was diluted in 100 μl Opti-MEM and mixed 1:1 with 100 μl Opti-MEM containing 5 μl Lipofectamine reagent. The mixture was incubated for 5 min at room temperature before adding dropwise to the well. Cells were incubated with the Lipofectamine for 4 to 5 h, then transfection medium was replaced with fresh culture medium or induction medium. Cells were incubated for a further 24 to 48 h before using in assays.

### Production of stable transfected cell lines

Various constructs based on hGPR35a, mGPR35, and rGPR35 were stably transfected into Flp-In TREx doxycyline-inducible 293 cells. The pcDNA5/FRT/TO vector containing the relevant cDNA was transfected into Flp-In TREx-293 parental cells using the FRT stable integration site. Cells were co-transfected with the relevant cDNA/pcDNA5/FRT/TO construct and the pOG44 Flp recombinase vector in a 1:8 ratio using PEI. After 48 h, cells were subcultured 1:10 and 1:30, and 24 h later, medium was changed to maintenance medium plus 200 μg/ml hygromycin to select for stable transfectants. Medium was changed every 3 days until individual colonies were visible by eye (10–14 days). Cells were then detached by incubating with trypsin-EDTA and pooled to give polyclonal cell lines which were maintained in hygromycin selection medium. When required, expression of the integrated gene was induced by addition of 100 ng/ml doxycycline for 18 to 24 h.

### Cell lysate preparation

Cell lysates were generated from HEK293 cells following transfection to express eYFP-fusion receptor constructs. Cells were harvested in ice-cold PBS and lysed in lysis buffer (150 mM NaCl, 50 mM Tris-HCl, 5 mM EDTA, 1% Nonidet P-40, 0.5% Na-deoxycholate, and 0.1% SDS), supplemented with protease and phosphatase inhibitor tablets on a rotating wheel for 30 min at 4 °C. Samples were then centrifuged for 15 min at 11,000*g* at 4 °C. Protein content was assessed using a BCA protein assay kit (Thermo Fisher).

### Receptor immunoprecipitation and immunoblotting

eYFP-linked receptor constructs were immunoprecipitated from 200 μl cell lysate using a GFP-Trap kit (Chromotek) according to manufacturer's instructions. Immunocomplexes were washed three times in wash buffer, resuspended in 100 μl Laemmli buffer, and incubated at 60 °C for 5 min. Following centrifugation at 2500*g* for 5 min, 20 μl of immunoprecipitated proteins were resolved by SDS-PAGE on NuPAGE Novex 4 to 12% Bis-Tris Gels (Thermo Fisher). Gels were run in NuPAGE MOPS SDS Running Buffer (Thermo Fisher) at 200 V for 50 min, and proteins were transferred from the gel onto nitrocellulose membrane using a wet transfer system. Following transfer, the nitrocellulose membrane was blocked using 5% bovine serum albumin (BSA) in Tris-buffered saline (TBS, 50 mM Tris-Cl, 150 mM NaCl, pH 7.6) for 1 h at room temperature on an orbital shaker. The membrane was then incubated with appropriate primary antibody in 5% BSA TBS supplemented with 0.1% Tween (TBS-T) overnight at 4 °C. Anti-pSer^300^/pSer^303^-hGPR35a and pSer^298^/pSer^301^-mGPR35 were diluted 1:1000, and anti-GFP was diluted 1:10,000. The membrane was washed (3 × 5 min with TBS-T) and incubated for 2 h at room temperature with IRDye 800CW anti-rabbit or anti-goat secondary antibody diluted 1:10,000 in 5% BSA TBS-T. After washing (3 × 5 min with TBS-T), proteins were detected using a LI-COR Odyssey imaging system according to the manufacturer's instructions.

### [^32^P] phospholabeling assay

Flp-In TREx-293 GPR35-HA cells were seeded in 6-well plates at 2 × 10^5^ cells/well and incubated overnight at 37 °C. GPR35-HA expression was induced by adding 100 ng/ml doxycycline and incubating overnight at 37 °C. Cells were washed three times with Krebs/Hepes buffer without phosphate and incubated in this buffer plus 100 μCi/ml [^32^P]-orthophosphate for 90 min at 37 °C. Cells were pretreated with antagonist for 5 min where stated, then stimulated for 5 min with vehicle only (dimethylsulfoxide) or agonist and immediately lysed by addition of 750 μl/well lysis buffer containing complete EDTA-free protease inhibitor cocktail and PhosSTOP phosphatase inhibitor cocktail. Lysates were incubated on ice for 5 min, then cleared by centrifugation at 20,000 ×*g* for 20 min at 4 °C. GPR35-HA variants were immunoprecipitated from the cleared lysates using anti-HA affinity matrix. Lysates were incubated with 50 μl affinity matrix suspension with rotation for 2 h at 4 °C. Immunocomplexes were collected by centrifuging at 1000*g* for 1 min and washed 3x with lysis buffer, subjecting to centrifugation and aspirating supernatant after each wash. GPR35-HA was eluted from the matrix by adding 50 μl Laemmli buffer, vortexing, and incubating at 65 °C for 10 min. Immunoprecipitants were separated by SDS-PAGE on 10% Bis-Tris acrylamide gels, which were dried and exposed to X-ray film overnight at −80 °C. A small amount of each sample was run in parallel on a separate gel, then transferred to PVDF membrane and immunoblotted for GPR35-HA. Bands were quantified by densitometry using ImageJ software.

### Ligand treatment

To assess agonist-dependent receptor phosphorylation, cells were serum starved for 1 h, then pretreated with vehicle or antagonist for 15 min at 37 °C in a 5% CO_2_ humidified atmosphere, then treated with vehicle or agonist for a further 5 min. After treatment, cells were put on ice and harvested in ice-cold PBS.

### Treatment with λ-PPase

To remove phosphate groups, immunocomplexes were treated with λ-PPase at a final concentration of 10 unit/μl for 90 min at 30 °C before elution with 2 × Laemmli buffer as described earlier.

### Cell treatment with compound 101

To inhibit GRK2/3 function, cells were treated with 10 μM compound 101 for 30 min at 37 °C in a 5% CO_2_ humidified atmosphere, prior to ligand treatment.

### Analysis of N-glycosylation

Endoglycosidase treatment was carried out overnight at 37 °C using peptide *N*-glycosidase F (NGaseF) at a final concentration of 1 unit/μl.

### Immunocytochemistry

Cells were seeded at 7.5 × 10^4^ cells/well on poly-D-lysine–coated 13 mm round coverslips in 24-well plates and maintained at 37 °C in a 5% CO_2_ humidified atmosphere. Cells were treated as described previously, then fixed with 4% (w/v) paraformaldehyde in PBS for 10 min at room temperature. Fixed cells were washed 3 × 5 min in TBS, then permeabilized with TBS + 0.1% saponin for 10 min at room temperature. Cells were then blocked for 1 h at room temperature in blocking buffer (TBS, 10% goat serum, and 1% BSA) before incubating with primary antibody (anti-pSer^300^/pSer^303^-hGPR35a, pSer^298^/pSer^301^-mGPR35 and anti-HA were diluted 1:400 in blocking buffer) overnight at 4 ^o^C. Subsequently, cells were washed 3 × 5 min in TBS, then incubated with secondary antibody (Alexa Fluor 488-goat anti-rabbit IgG and Alexa Fluor 488-donkey anti-rat IgG 1:400 dilution in blocking buffer) for 1 h at room temperature. Cells were washed 3 × 5 min in TBS, and coverslips were mounted onto glass slides using VECTASHIELD Mounting Medium with DAPI (Vector laboratories). Images were taken using a Zeiss LSM 880 confocal equipped with a 63x/1.4 NA Plan Apochromat oil-immersion objective.

### Arrestin-3 recruitment BRET assays

BRET-based arrestin-3 recruitment assays were used to assess the effect of C-terminal tail mutations on arrestin-3 interactions with forms of GPR35. HEK293T cells were seeded in 10 cm^2^ dishes and transiently co-transfected with WT or mutant forms of orthologues of GPR35-eYFP, each with a FLAG epitope tag engineered into the N-terminal domain, and arrestin-3 fused to *Renilla* luciferase (arrestin-3-RLuc) in a 4:1 ratio using PEI. Control cells were transfected with arrestin-3-RLuc only. After 24 h, cells were detached by incubating with trypsin-EDTA and seeded at 6 × 10^4^ cells/well in poly-D-lysine coated white 96-well plates, then incubated overnight at 37 °C. Cells were washed once with prewarmed (37 °C) Hanks' buffered saline solution (HBSS) and incubated in HBSS for 30 to 60 min at 37 °C. During incubation, the eYFP signal (excitation 485 nm, emission 520 nm) was read on a PHERAstar FS (BMG Labtech) to estimate relative receptor expression. The RLuc substrate coelenterazine-h (Promega) was added to a final concentration of 5 μM, and the plate incubated for 10 min at 37 °C protected from light. Agonists were added at the relevant concentrations in triplicate, and the plate incubated for a further 5 min at 37 °C, then the emissions at 475 nm and 535 nm were read on a PHERAstar FS. Net BRET values were obtained by dividing the emission at 535 nm by the emission at 475 nm and subtracting the 535 nm/475 nm ratio for cells expressing only the arrestin-3-RLuc donor (the basal BRET): Net BRET = (em535 nm/em475 nm) – (em535 nm/em475 nm [RLuc only]).

### Cell surface enzyme-linked immunosorbent assay

Cell surface expression of receptors was quantified by live-cell ELISA. Cells were seeded at 6 × 10^4^ cells/well in poly-D-lysine–coated clear 96-well plates and incubated overnight at 37 °C. Cells were incubated with primary antibody (mouse monoclonal anti-FLAG M2 1:1000) in culture medium for 30 min at 37 °C, then washed once with DMEM–Hepes and incubated with secondary antibody (horseradish peroxidase–sheep anti-mouse IgG 1:5000) and 10 μg/ml Hoechst 33342 in culture medium for 30 min at 37 °C protected from light. Cells were then washed twice with warmed (37 °C) PBS. During the second wash, the Hoechst 33342 signal (excitation 355 nm, emission 460 nm) was read on a POLARStar Omega (BMG Labtech). Finally, PBS was removed, and 100 μl/well room temperature TMB substrate was added. The plate was incubated for 5 min at room temperature protected from light, then the absorbance at 620 nm was read on a POLARStar Omega. Absorbance was corrected for cell number by dividing by the Hoechst 33342 signal.

### Mass spectrometry

Mass spectrometry was performed at the University of Leicester Proteomics Facility. Samples were analyzed as described previously (34). LC-MS/MS was carried out using an LTQ Orbitrap mass spectrometer (Thermo Fisher Scientific). A reverse-phase trapping column (0.3 mm inner diameter x 1 mm) containing 5 μm C18 300 Å Acclaim PepMap medium (Dionex) was loaded with the tryptic peptides at high flow rate. Peptides were eluted through a reverse phase capillary column (75 μm inner diameter x 150 mm) containing Symmetry C18 100 Å medium (Waters) that was self-packed using a high-pressure packing device (Proxeon Biosystems) (34).

### Database searching

All MS/MS samples were analyzed using Mascot (Matrix Science; version 2.2.04) and X! Tandem (The GPM, thegpm.org; version CYCLONE [2010.12.01.1]). Mascot and X! Tandem were searched with a fragment ion mass tolerance of 0.020 Da and a parent ion tolerance of 10.0 PPM. The UniprotHuman_2013_08 database (88,378 entries) was searched. Carbamidomethyl of cysteine was specified as a fixed modification and Glu->pyro-Glu of the n-terminus, ammonia-loss of the n-terminus, gln->pyro-Glu of the n-terminus, oxidation of methionine, and phosphorylation of serine, threonine, and tyrosine were specified in X! Tandem as variable modifications. Oxidation of methionine and phospho of serine, threonine, and tyrosine were specified in Mascot as variable modifications.

### Criteria for peptide identification

Scaffold (version Scaffold_4.8.7, Proteome Software Inc) was used to validate MS/MS-based peptide and protein identifications. Peptide identifications were accepted if they could be established at greater than 20.0% probability. Peptide probabilities from X! Tandem and Mascot were assigned by the Scaffold Local FDR algorithm. Peptide probabilities from X! Tandem were assigned by the PeptideProphet algorithm (56) with Scaffold delta-mass correction. Protein identifications were accepted if they could be established at greater than 95.0% probability and contained at least two identified peptides. The mass spectrometry proteomics data have been deposited to the ProteomeXchange Consortium *via* the PRIDE partner repository (57) with the dataset identifier PXD030548 and 10.6019/PXD030548.

### Receptor internalization

Receptor internalization was qualitatively examined using live-cell confocal microscopy. Cells were seeded at 0.5 to 1 × 10^5^ cells/well on poly-D-lysine–coated 30 mm round coverslips in 6-well plates and incubated for at least 48 h at 37 °C to recover normal morphology after seeding. For transient transfection, cells were transfected using Lipofectamine 24 h after seeding. Cells were washed once with HBSS, and coverslips were placed in a microscope chamber containing HBSS. eYFP images were taken before treatment and at 15 min intervals following addition of agonist. Images were acquired on a ZEISS Axio Observer.Z1 microscope fitted with a spinning disk structured illumination VivaTome, using narrow band 490/20 nm excitation and 536/40 nm emission and a 63× oil-immersion Plan-Apochromat objective, and captured on an Axiocam MRm charge-coupled device camera. Images were optically sectioned using AxioVision software (ZEISS). Receptor internalization was quantitatively assessed using ArrayScan high content analysis. Cells were seeded at 4 × 10^4^ cells/well in poly-D-lysine–coated black-walled, clear-bottomed 96-well plates and incubated overnight at 37 °C. Culture medium was replaced with serum-free medium containing agonist concentrations in triplicate. Cells were incubated with agonist for 45 min, then washed once with PBS, and fixed with 3.7% (w/v) paraformaldehyde for 1 h at room temperature. Fixed cells were washed three times with PBS, then stained with 10 μg/ml Hoechst 33342 for 30 min at room temperature protected from light. Cells were washed 3× with PBS before acquiring eYFP and DAPI images using a Cellomics ArrayScan II high content imager. Internalization was quantified using an algorithm designed to identify the number of individual ‘endosomal recycling compartments’ in the eYFP channel, which was normalized against cell number using the Hoechst 33342 signal in the DAPI channel.

### Statistical analysis

All data are presented as means ± SD of at least three independent experiments and as appropriate as scatterplots with individual experiments recorded. Comparisons were assessed by one-way ANOVA with Tukey’s multiple comparisons posttest where appropriate. Data analysis and curve fitting were carried out using the GraphPad Prism software package, version 8 (GraphPad).

## Data availability

All data are freely available from the communicating author (Graeme.Milligan@glasgow.ac.uk). The mass spectrometry proteomics data have been deposited to the ProteomeXchange Consortium *via* the PRIDE [36] partner repository with the dataset identifier PXD030548 and 10.6019/PXD030548.

## Supporting information

This article contains supporting information.

## Conflict of interest

S. S. is founder and shareholder of 7TM Antibodies GmbH, Jena, Germany. The other authors declare no conflict of interests.
