# Supplementary material for: Agonist-induced phosphorylation of orthologues of the orphan receptor GPR35 functions as an activation sensor
Source: J Biol Chem. 2022 Jan 29;298(3):101655. doi: 10.1016/j.jbc.2022.101655 (PMC8892012; doi:10.1016/j.jbc.2022.101655)
Supplement: Supplemental Figures S1–S6 and Table S1 [file mmc1.docx]

**Agonist-induced phosphorylation of orthologues of the orphan receptor GPR35 functions as an activation sensor**

**Nina Divorty1,2, Laura Jenkins1, Amlan Ganguly1,**

**Adrian J. Butcher3, Brian D. Hudson1, Stefan Schulz4-5,**

**Andrew B. Tobin1, Stuart A. Nicklin2, and Graeme Milligan1***

1. The Centre for Translational Pharmacology, Institute of Molecular, Cell and Systems Biology, College of Medical, Veterinary and Life Sciences, University of Glasgow, Glasgow G12 8QQ, United Kingdom
2. Institute of Cardiovascular and Medical Sciences, College of Medical, Veterinary and Life Sciences, University of Glasgow, Glasgow G12 8QQ, United Kingdom
3. Department of Clinical Neurosciences, University of Cambridge, Cambridge CB2 0AH, United Kingdom
4. 7TM Antibodies GmbH, Hans-Knöll-Str. 6, 07745 Jena, Germany
5. Institute of Pharmacology and Toxicology, University Hospital Jena, Drackendorfer Str. 1, 07747 Jena, Germany

# *Address correspondence to Graeme Milligan (Graeme.Milligan@glasgow.ac.uk)

**Figure S-1**


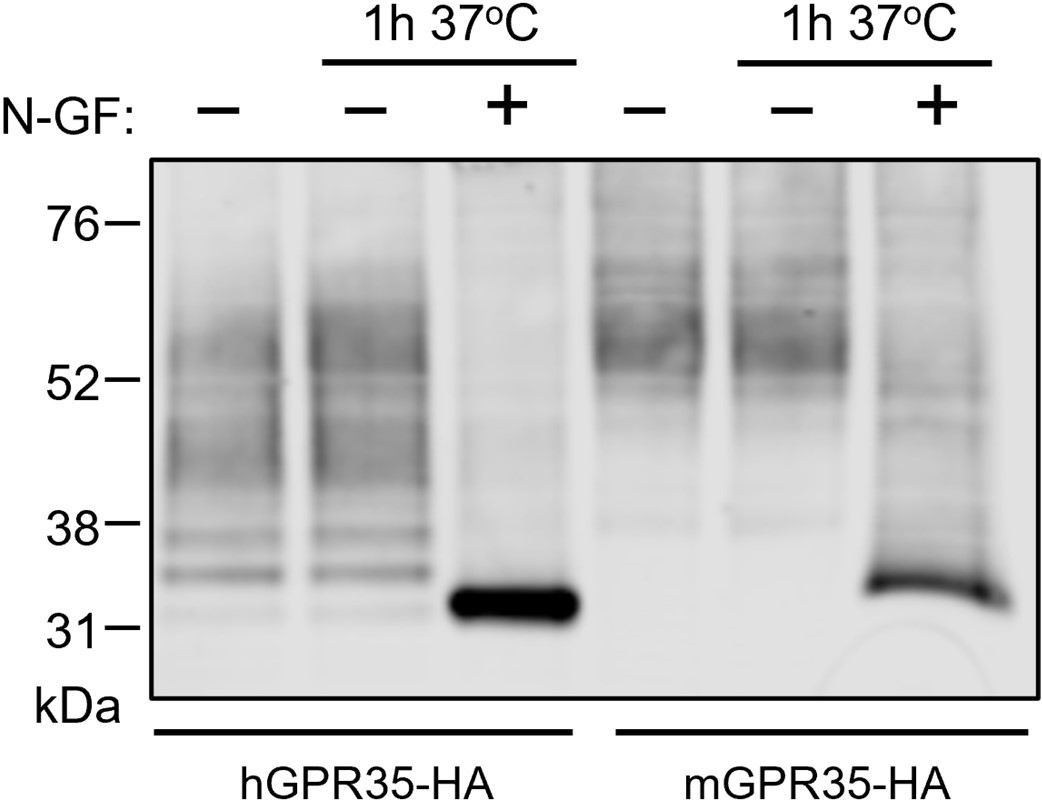


**Figure S-1**: **Human and mouse GPR35-HA are N-glycosylated**

Representative anti-HA immunoblots of hGPR35a-HA and mGPR35-HA-expressing HEK293 cell lysates in their native states or following treatment with N-glycosidase F (N-GF = *N*-glycosidase F).

**Figure S-2**


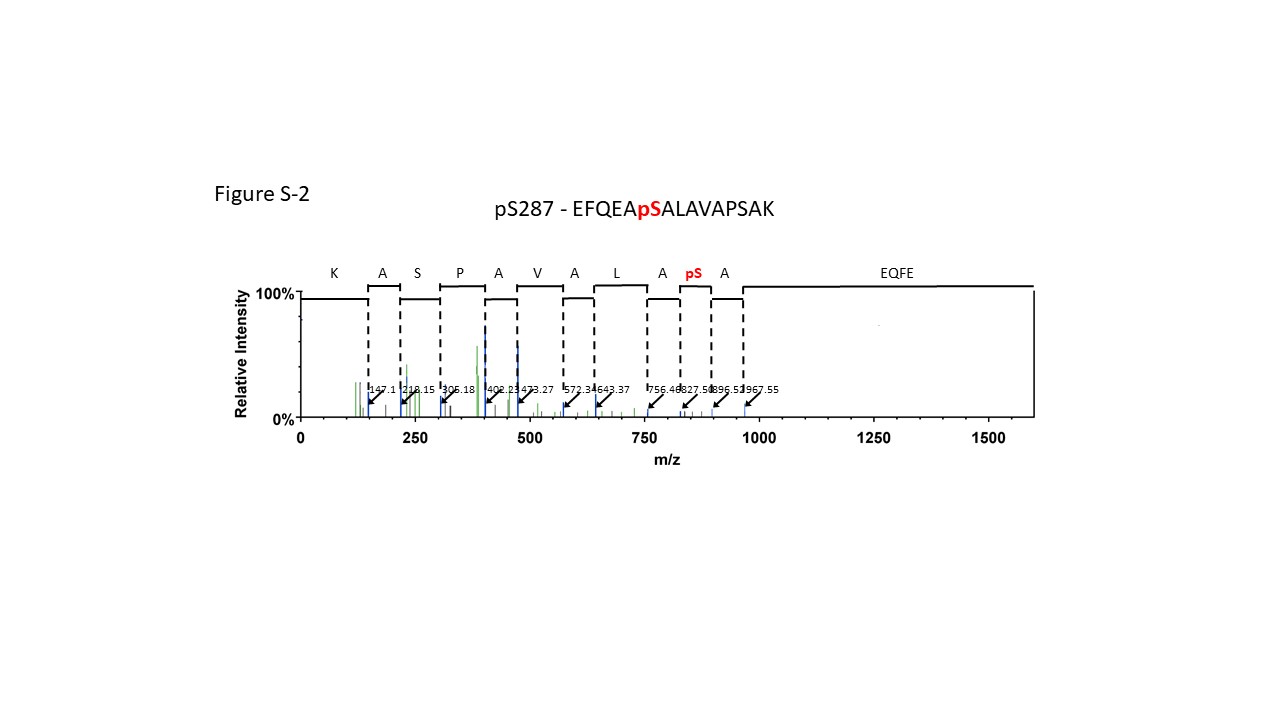


**Figure S-3**

**
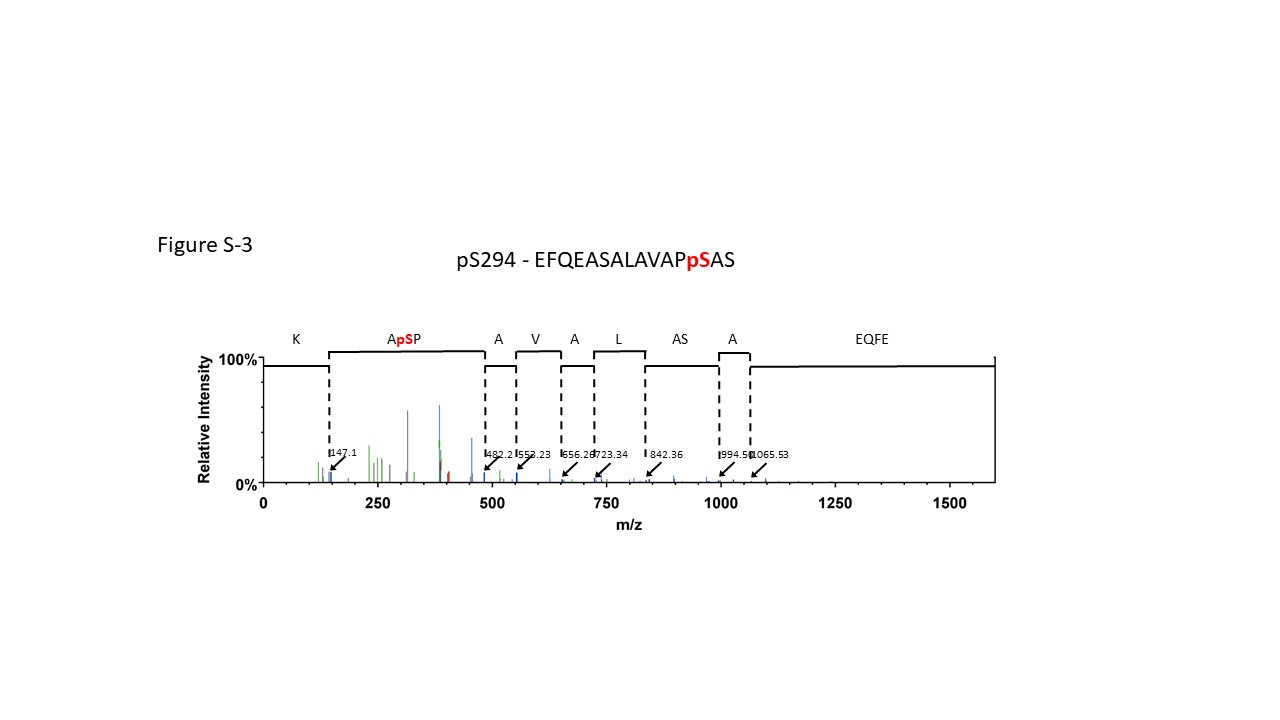
**

**Figure S-4
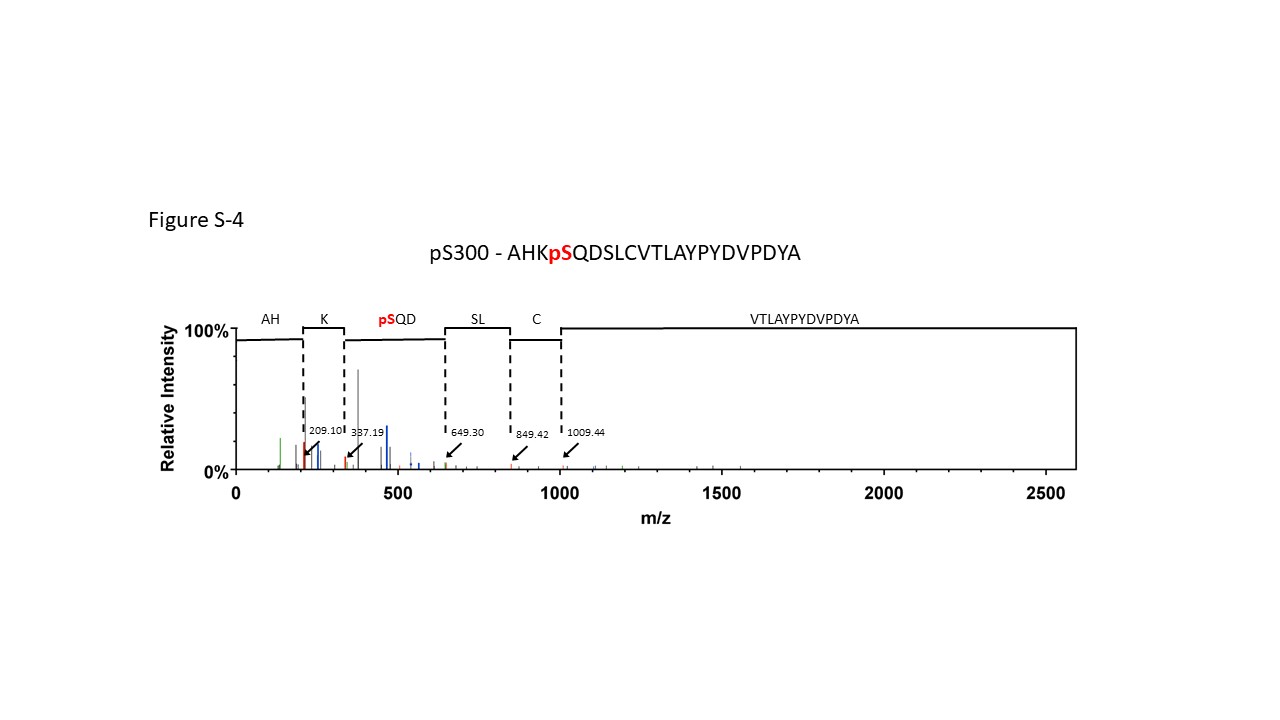
**

**Figure S-5**


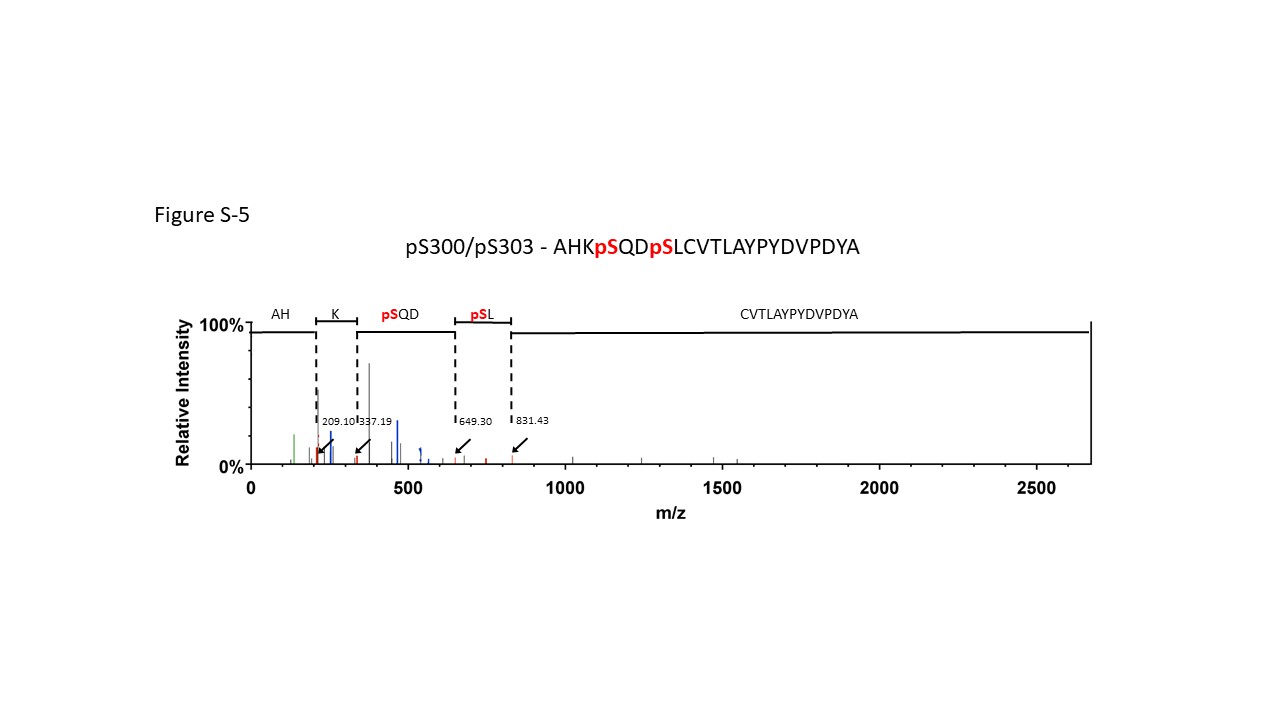


**Figure S-6**


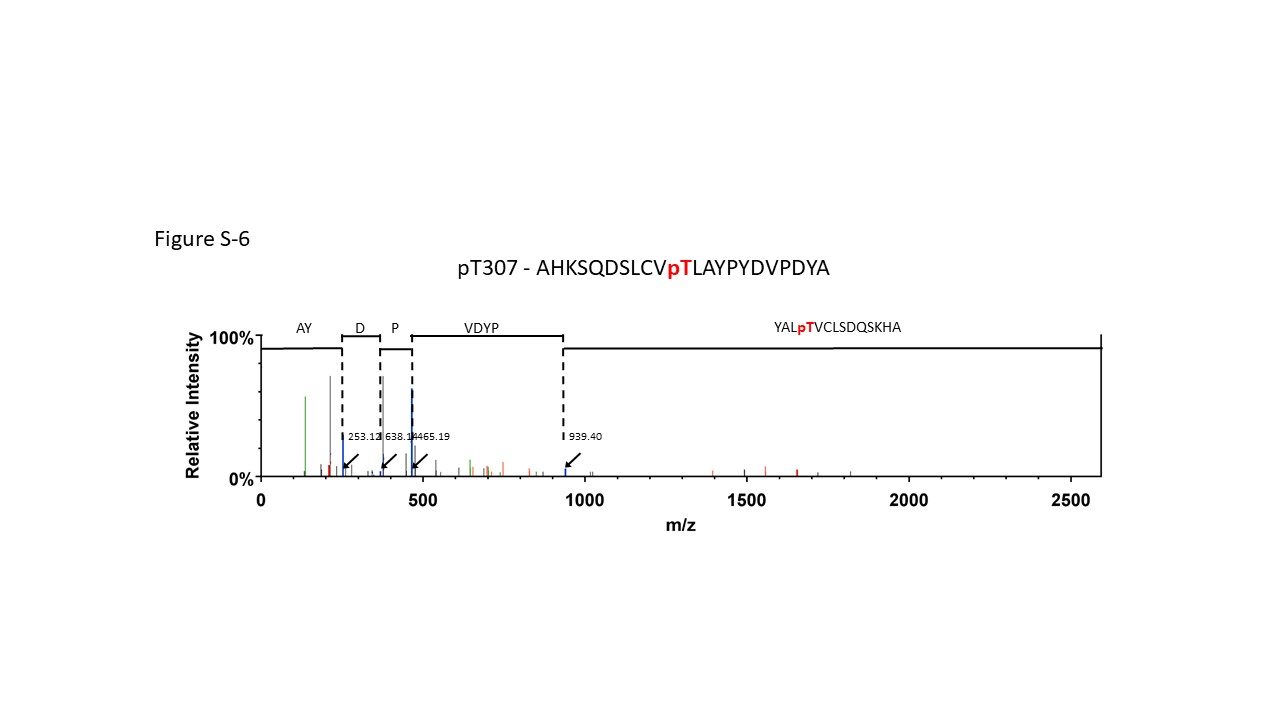


**Figures S-2 to S-6. Details of mass spectrometry studies**

These Figures provide details of mass spectrometry studies on zaprinast-activated hGPR35a-HA that are summarized in Figure 2 of the main text and detailed in Experimental Procedures. Further details are available at the ProteomeXchange Consortium via the PRIDE partner repository with the dataset identifier PXD030548 and 10.6019/PXD030548.

**Table S-1**

| **Phosphopeptide sequence** | **Precursor**  **ion charge** | **m/z** | **Mascot peptide**  **identity score** |
| --- | --- | --- | --- |
| (K)EFQEASALAVAP**pS**AK(A) | 2+ | 799.88 | 28.4 |
| (K)EFQEA**pS**ALAVAPSAK(A) | 2+ | 799.88 | 28.3 |
| (K)AHK**pS**QD**pS**LCVTLAYPYDVPDYA(-) | 2+ | 1337.06 | 30.4 |
| (K)AHKSQDSLCV**pT**LAYPYDVPDYA(-) | 3+ | 865.05 | 31.0 |
| (K)AHK**pS**QDSLCVTLAYPYDVPDYA(-) | 2+ | 1297.07 | 31.1 |
| (K)AHKSQD**pS**LCVTLAYPYDVPDYA(-) | 3+ | 865.05 | 31.0 |

Zaprinast-induced phosphorylation of amino acids with the intracellular C-terminal domain of hGPR35a-HA characterized identified by mass spectrometry.
